# Electrocatalysis on the nm scale

**DOI:** 10.3762/bjnano.6.103

**Published:** 2015-04-21

**Authors:** R Jürgen Behm

**Affiliations:** 1 Institute of Surface Chemistry and Catalysis, Ulm University, Albert-Einstein-Allee 47, D-89081 Ulm, Germany

**Keywords:** electrocatalysis

The past two decades have seen a renewed and rapidly growing interest in the fields of electrochemistry and electrocatalysis. This is on the one hand stimulated by applications in energy conversion and energy storage, where highly efficient electrochemical/electrocatalytic processes are considered to be an indispensable part of modern energy concepts based on the use of renewable energy sources. However, it is also pushed by the rapid development of modern in situ spectroscopy and microscopy tools, such as in situ vibrational spectroscopy, in situ X-ray spectroscopy/diffraction, and in situ scanning probe microscopy, just to name a few, as well as the enormous progress that has been made in the theoretical description of processes occurring at the electrochemical solid–liquid interface. The experimental methods provide, at least in principle, the opportunity to gain insight into the processes occurring at the solid–electrolyte interface on an unprecedented, atomic/molecular level. The theory has not only developed to a stage where a reliable description of complex surface structures and surface processes (at the solid–gas interface) is possible based on first-principles electronic structure theory (in particular, (periodic) density functional theory (DFT)), but it is also increasingly developing new approaches for a more realistic modeling of the electrochemical solid–liquid interface from first principles. Although there is still a long way to go, it is not unrealistic to assume that an atomic/molecular scale understanding of the elementary processes occurring at the electrochemical interface (similar to that developed for the solid–gas interface) is within reach, given the rapid progress in both theory and experiment. Furthermore, the employment of modern strategies from nanotechnology for the systematic fabrication of optimized, nanostructured electrodes and electrocatalysts sets the stage and provides the means for a systematic, knowledge-based optimization of electrochemical and electrocatalytic processes. This is unprecedented in this discipline and will hopefully allow us to satisfy the expectations from the technology side.

These aspects are the topics of the present Thematic Series, “Electrocatalysis on the nm scale”. This work was initiated by and is largely based on a workshop organized by the Research Group, “Elementary Reaction Steps in Electrocatalysis: Theory meets Experiment”, which was held at Reisensburg Castle (close to Ulm, Germany) in 2013. As suggested by the name of the Research Group, the topics equally cover experiment and theory. They range from fundamental aspects of electrochemistry, such as anion adsorption [1] or potential induced restructuring of the electrode surface [2], to the presentation of new theoretical concepts, the mechanistic understanding [3], and the theoretical description of important electrocatalytic reactions, such as hydrogen evolution/water splitting [4–5] or electrocatalytic ammonia synthesis [6]. Additionally, mechanistic studies of electrocatalytic reactions, such as O_2_ reduction [7], CO oxidation [8] or the electrooxidation of small organic molecules [9], are presented. The potential of in situ microscopy [10] and in situ spectroscopy [8–9], in addition to electrochemical measurements for the characterization of electrode surfaces/electrocatalysts and for the mechanistic understanding of electrocatalytic reactions is illustrated. Finally, the systematic use of nanostructuring strategies is outlined, both for fundamental studies [8] and for the directed design of active and stable Pt/C fuel cell catalysts [11]. In summary, despite the limited number of contributions to this Thematic Series, it provides a broad perspective covering a variety of important topics in modern electrocatalysis research, with a clear focus on the nanoscale understanding of the relevant processes.

I would like to thank all authors for their excellent contributions, as well as the referees for their valuable and prompt reports. Special thanks go to the team at the Beilstein Journal of Nanotechnology for their continuous support in the handling of this series. Finally, I would like to acknowledge the Beilstein-Institut for its support of open access policies, allowing colleagues from all over the world to freely access the contributions in this journal, as well as the financial support from the Deutsche Forschungsgemeinschaft (FOR 1376) for the initiating workshop and for part of the work.

R. Jürgen Behm

Ulm, March 2015
